# Complete genome sequences of *Lactobacillus acidophilus* strain P42 and *Limosilactobacillus reuteri* strain P43 isolated from chicken cecum

**DOI:** 10.1128/mra.01140-23

**Published:** 2024-03-19

**Authors:** Hosni M. Hassan, Mary Mendoza, Allison N. Dickey

**Affiliations:** 1 Prestage Department of Poultry Science, North Carolina State University, Raleigh, North Carolina, USA; 2 Bioinformatics Research Center, North Carolina State University, Raleigh, North Carolina, USA; University of Maryland School of Medicine, Baltimore, Maryland, USA

**Keywords:** complete genome, PacBio HiFi reads, *L. acidophilus *P42, *L. reuteri*P43, poultry

## Abstract

The gut microflora contains a diverse microbial population that is influenced by the host and the environment. We report the complete circular genome sequences of *Lactobacillus acidophilus* strain P42 and *Limosilactobacillus reuteri* strain P43 isolated from chicken cecal samples. P42 and P43 could potentially serve as poultry probiotic strains.

## ANNOUNCEMENT

The gut microbiome is important for host immune system modulation and for protection from pathogens (1). We previously published a draft genome of *Limosilactobacillus reuteri* P43 (MCNS00000000) that was assembled from Illumina MiSeq short reads (2). In this report, we sequenced a new isolate, *Lactobacillus acidophilus* P42, and re-sequenced P43 using PacBio HiFi reads to generate single chromosomal contigs. P42 and P43 were isolated from the cecal microbiota of 4-week-old female commercial white leghorn (egg-layer) chickens (W-36, Hy-line North America, Mansfield, GA) fed a standard diet containing 1% Oligomate 55 [galacto-oligosaccharides (GOS)] from Yakult Pharmaceutical Industry (Japan) (2, 3). The birds were euthanized according to a protocol (15-065-A) approved by the Institutional Animal Care and Use Committee at North Carolina State University (OLAW D16-00214). The isolates were selected based on their morphology (G^+^, short rods) and their ability to ferment lactose. P42 and P43 were grown anaerobically (Coy anaerobic chamber) for 24 h at 37°C in MRS media, and genomic DNA was isolated using the Promega Wizard Genomic DNA Purification Kit (Promega Corporation, Madison, WI). HiFi reads were generated on a PacBio Sequel IIe. Library preparation and barcoding included use of the SMRTbell Prep Kit 3.0 with the SMRTbell Barcoded Adapter Plate 3.0. The DNA was sheared via Covaris g-TUBE to ~16–17 kb. AMPure PB bead size selection was used to remove fragments < 5 kb.

The number of HiFi reads for P42 was 809,055 (N50:10,089) and for P43 was 1,514,625 (N50:10,851). The reads were subsampled before assembly using seqtk -s10 (v.1.3) (4) to 26,149 reads for P42 and to 24,128 reads for P43. P42 was assembled using hifiasm (v.0.19.3-r572) (5) with the --primary flag which resulted in one circular chromosome. P43 was assembled using Canu (v.2.2) (6) with the genomeSize = 2.0 m and the pacbio-hifi flags. The P43 assembly included eight contigs, one of which was retained as the chromosomal contig. Of the seven removed contigs, six were identified as likely bubbles and one was formed from few reads. MUMmer nucmer --maxmatch --nosimplify (v.4.0.0rc1) (7) was used to identify overlapping regions between the chromosomal contig start and end (overlap: 6,450–17,322 bp and 2,154,692–2,165,563 bp). For circularization, the end of the contig was removed between 2,148,243 and 2,165,563 bp. Because the contig start overlap region did not begin at the first bp, 6,450 was subtracted from 2,154,692 to obtain the circularized contig end point. The final assembly summaries are shown in (Table 1).

**TABLE 1:** Assembly summary for P42 and for P43

Strain	GenBank accession numbers	Chromosome size (bp)	GC%	Mean read coverage	Gene number	rRNAs, tRNAs, ncRNAs	Assigned species
P42	CP136510	1,991,561	34.7	125x	1,924	*12, 61, 3*	*Lactobacillus acidophilus*
P43	CP136906	2,148,242	38.8	116x	2,077	*18, 70, 3*	*Limosilactobacillus reuteri*

The origin for the circularized chromosomes was manually set to the dnaA gene on the forward strand. Genome annotations were generated with the NCBI PGAP (v.6.6) (8). BBMap SendSketch (v.38.90) (9) was used to assign species. The top hit for P42 was *Lactobacillus acidophilus La-14* (ANI:100.00%), and the top hit for P43 was *Limosilactobacillus reuteri subsp. murium* (ANI:98.17%).

## Data Availability

The assemblies were deposited in GenBank under accession numbers CP136510 (P42) and CP136906 (P43). The HiFi reads were deposited in SRA under accession numbers SRR26302841 (P42) and SRR26302840 (P43)
